# Introducing AfroGrid, a unified framework for environmental conflict research in Africa

**DOI:** 10.1038/s41597-022-01198-5

**Published:** 2022-03-29

**Authors:** Justin Schon, Ore Koren

**Affiliations:** 1 Senior Research Analyst, Policy Analysis Unit, AidData, College of William & Mary, Williamsburg, Virginia USA; 2 Department of Political Science, Indiana University, Bloomington, Indiana USA

**Keywords:** Climate-change policy, Developing world, Governance

## Abstract

In this study, we present Afro-Grid: an integrated, disaggregated 0.5-degree grid-month dataset on conflict, environmental stress, and socioeconomic features in Africa covering 1989–2020, intended to propel research on these issues forward. Afro-Grid offers several important extensions for researchers and policymakers, including: (i) standardizing (using established methods) data sources on conflict, environmental stress, and socio economic factors across spatial and temporal scales; (ii) combining these data into a single, openly-available file, maximizing the accessibility of these data for researchers and policymakers regardless of their software background; and (iii) including NDVI and dual-series harmonized night lights series that have traditionally not been accessible to researchers without advanced computational expertise. Using a series of comparative regressions at the grid-month and grid-year levels, combined with reporting descriptive statistics and visualizations, we illustrate that this temporally and geographically disaggregated dataset provides valuable extensions for research related to the climate-conflict nexus and the role of socioeconomic features in shaping conflict trends, as well as for research and data-driven policy on development and conflict.

## Abstract (summary)

**Table:** 

Measurement(s)	Conflict • climate • Development
Technology Type(s)	Remote sensing and computational analysis
Factor Type(s)	NDVI • Temperature • Precipitation • SPEI • Nighttime light emissions • Population densities
Sample Characteristic - Organism	Homo sapiens
Sample Characteristic - Environment	geographic feature
Sample Characteristic - Location	Africa

## Background & Summary

Quantitative, mixed methods, and even qualitative social scientists increasingly rely on high resolution data to study local-level political and economic development. This is especially true for environmental conflict analysis, which examined the impact of temperature^1,2^, precipitation^3–5^, food (in)security^6–8^, water (in)security^9^, and environmental migrants^10,11^, among others. While earlier work relied on country level indicators, quantitative and mixed methods researchers turn to geographically disaggregated data to capture subnational conflict dynamics^12,13^. Notable efforts in this regard include the Armed Conflict Location and Event Dataset (ACLED)^14^, UCDP’s Geographic Event Data dataset (GED)^15^, the PITF’s Worldwide Atrocity Dataset (WAD)^16^, and the Social Conflict in Africa Dataset (SCAD)^17^. In addition to timing and location, these event datasets disaggregate violence by type (e.g., armed conflict, riots and protests) and actor (rebels, governments, civilians). An important extension is PRIO-GRID, a vector grid network with a resolution of 0.5 × 0.5 decimal degrees that includes dozens of relevant indicators measured at annual and less frequent time scales^13^, which has become popular among conflict analysts, and researchers of development, economics, and politics broadly.

Despite these advantages, extant standardized geographic datasets have limitations. The information included is – often – more than half a decade old. Some datasets also incorporate variables that do not vary over time, or that vary annually or over multiple years^13^. Conflict trends may be heavily influenced by seasonal environmental changes (e.g., in rainfall and temperature) rather than by annual differences in their averages. Relying on the year as the temporal unit of analysis may therefore provide an inaccurate picture of monthly dynamics^18^. Additionally, often-used measures of temperature, precipitation, droughts, and floods are less-than-effective proxies of environmental stress’ impacts and can be empirically problematic^9,19–21^. Civilians can adapt to variations over time, switching to non-water-intensive crops or less demanding livestock^22^. Similarly, while research has become increasingly reliant on nighttime light emissions to measure local-level development^23,24^, shifts in data sources prevent researchers from using night lights data for time periods covering before and after 2013.

AfroGrid presents an important extension to spatio-temporal socioeconomic datasets, with several implications for conflict research and beyond. First, we standardize (using established methods) multiple new data sources on conflict, environmental stress, and socio-economic factors across spatial and temporal scales, which improve – in terms of both temporal availability and empirical quality – on geographic datasets such as PRIO-Grid. AfroGrid integrates these variables into 0.5-degree resolution grid cells (i.e., geographic squares of about 60KM × 60KM at the equator, which decrease in size as one moves toward the poles) for the entire African continent (a total of 10,674 cross-sectional grid cells) over the 1989–2020 period, with information recorded at the monthly (or annual, for some variables) level for all periods and locations where it is available.

Second, these data have been combined into a single, openly-available csv file, maximizing these data’s accessibility for end users regardless of their software background. To allow researchers to integrate their own data, AfroGrid includes month and year indicators, latitude and longitude for each cell’s centroid, and PRIO-GRID^13^ and Correlates of War (COW) country IDs^25^. Third, the NDVI and dual-series harmonized night lights (discussed in detail below) have traditionally not been accessible to researchers without advanced computational expertise. Only a limited number of tools allow for harmonizing and synthesizing these data, especially when open-source resources are concerned.

These features mean that AfroGrid’s contribution goes beyond the fast-growing field of environmental conflict research. Environmental and conflict stressors impact a host of political and socioeconomic development indicators within African countries. Easily-accessible data can inform multiple bodies of research on these issues. Accordingly, we offer AfroGrid as a supplement to facilitate more detailed and spatially sensitive analyses of disaggregated political, economic, and social phenomena. Finally, in providing standardized data to answer questions on development and conflict, these tools can inform not only research, but – considering the increased reliance of policy analysis on Big Data tools – also policymaking on these topics.

## Methods

### Measuring environmental variability and development

#### NDVI, precipitation, temperature, and drought

A key contribution of this data note is in standardizing several measures of environmental variations and stress in an easily-accessible data format. In this regard, we first incorporate into AfroGrid the Normalized Difference Vegetation Index (NDVI), a remote sensing measure of greenery (e.g., grassland, agricultural productivity). Information for coding AfroGrid’s NDVI indicator comes from NASA’s MODIS Terra satellite system, which measures NDVI at the 0.08-degree (1 km) pixel level globally for each month between 1992 and 2018. We extracted these NDVI data with the MODIStsp R package^26^. Although there are several resources for researchers to obtain NDVI measurements, NASA’s MODIS satellites are some of the most widely used^21^. For all land areas, the NDVI value for each pixel range from 0 (no vegetation) to 1 (fully vegetated), although cells with water coverage can receive negative values (ranging from −1 to 0).

We standardize NDVI data to the 0.5-degree cell-month, creating three NDVI indicators. We first average NDVI values over all 0.08 pixels within a given grid cell during a given month to create an *NDVI (mean)* indicator. We then aggregate two additional NDVI-based indicators by taking the monthly values from the pixels with the highest and lowest, respectively, to the cell-month level to create *NDVI (max)* and *NDVI (min)*. Because they vary by month for each grid cell, such indicators provide a major advantage over measures such as cropland coverage used in past research (e.g^6,27^.), which are time constant. They also provide a useful extension considering the potential problems involved in using proxies such as precipitation highlighted in Background section as well as regarding the precision of temperature measurements, especially in conflict-afflicted zones^28^. These grid-month indicators are therefore especially useful for researching seeking to capture seasonal variations in greenery in partly-arid areas or quantify the risk of desertification and the resulting impacts in such locations.

Nevertheless, in addition to these NDVI-based measures, we also operationalize indicators of temperature, precipitation, and drought using information from the CRU TS monthly high-resolution gridded multivariate climate dataset (Version 4)^29^. We first create two indicators, *Temperature (mean)* and *Precipitation (mean)* using the same method as used in creating *NDVI (mean)*, that is, by aggregating the monthly information for each measure and dividing it by the total number of pixels within a given grid cell. We then construct two additional indicators – *Temperature (anomaly)* and *Precipitation (anomaly)* – measuring monthly anomalies in these phenomena. To create both measures, we calculate rolling 30-year *Z*-scores in each month for a particular grid cell using the zoo package in R. For example, for grid cell *i* in May of 2010, we calculate the *Z*-score of temperature and precipitation in the same cell *i* for all Mays in the past 30 years. Higher recorded anomalies therefore indicate that the temperature or precipitation values in a given cell-month are an X number of standard deviations above/below their 30 year mean. This anomaly calculation allows users – in effect – to observe whether climate in a given month during a given year is unusual for that particular time of the year. It was performed on the full 1901–2020 dataset before being merged into Afro-Grid, allowing us to have anomaly measurements (our Temperature (anomaly) and Precipitation (anomaly) indicators) for the full temporal duration and geospatial spread of our data. The raw correlations between *NDVI (mean)* on the one hand, and *Temperature (mean)* and *Precipitation (mean)* on the other, are r = −0.08 and r = 0.67, respectively, illustrating that ‘indirect’ measures of environmental stress only partially (or not at all) reflect ‘direct’ measures of productivity.

Our drought indicator is the Standardized Precipitation Evapotranspiration Index (SPEI). This SPEI measure is calculated based on the 0.5 by 0.5 degree grid cell temperature and precipitation data in the CRU TS monthly gridded data^30–32^. Following our method for operationalizing temperature, precipitation, and NDVI, we calculate the mean SPEI value for each grid cell in each month. SPEI is a drought indicator that calculates the difference between precipitation and the potential evapotranspiration (PET) index for a given time scale. Potential evapotranspiration accounts for the role of temperature in evaporating water, so SPEI incorporates both precipitation and temperature. Positive values suggest that a location is receiving more water than it is using, whereas negative values suggest that water supply is not meeting water use. The SPEI measure we use is at the one-month time scale. SPEI measures are standardized and comparable across spatial and temporal scales.

**Fig. 1:**
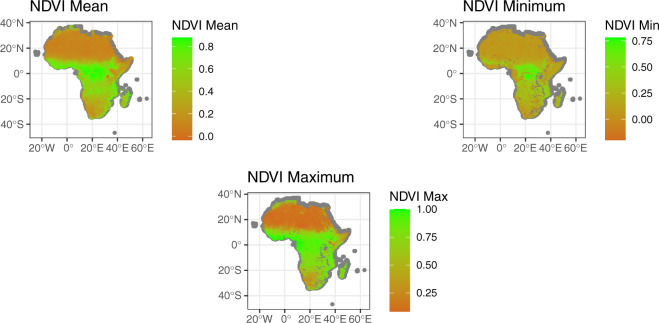
NDVI (mean), NDVI (min) and NDVI (max) AfroGrid values for June 2011.

**Fig. 2:**
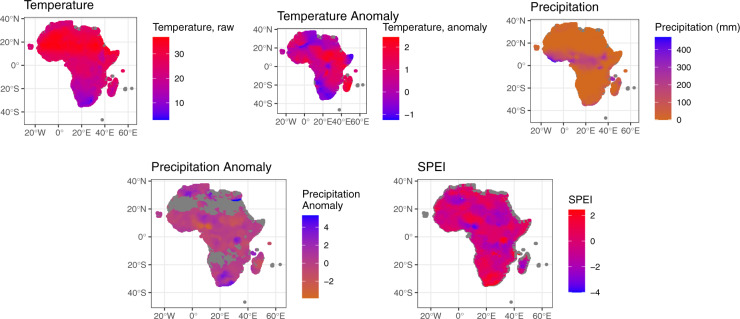
Temperature (mean), Precipitation (mean), Temperature (anomaly), Precipitation (anomaly), and SPEI AfroGrid values for June 2011.

For illustration, we plot values for each of our four NDVI variables for a randomly selected month (June 2011) in Fig. 1. Figure 2 then reports values for *Temperature (mean)*, *Precipitation (mean)*, *Temperature (anomaly)*, *Precipitation (anomaly)*, and SPEI drought. As Fig. 1 illustrates, *NDVI (mean)* is useful for measuring vegetation coverage, while the *NDVI (min)* and *NDVI (max)* allow researchers to capture the degree of monthly variability in terms of vegetation locally.

Moving to Fig. 2, the first two plots from the left on top show average temperature and temperature anomaly values across the continent for June 2011. Moving to the next top plot and the first bottom plot on the left, precipitation levels in June 2011 were relatively low across the continent, excluding the rain forest belt, which receives relatively high precipitation. In terms of anomalies, rainfall levels for each grid cell show important nuances regarding the locations of precipitation abundance or deficit. Finally, note that SPEI levels are generally in line with both temperature and precipitation levels for the same month.

#### Nighttime light and population

In addition to environmental factors, AfroGrid also includes data on nighttime light emissions and population densities within a given grid cell, which are often used in subnational studies (e.g^13,23,24,33^.). Note that unlike the rest of the indicators included in AfroGrid, which are all available at the cell-month level, these indicators were operationalized at the cell-year level, considering data availability and their slow-moving nature.

With respect to nighttime light, the most-often used nighttime light indicators rely on the Defense Meteorological Satellite Program (DMSP)/Operational Linescan System (OLS). This series is available only up to 2013, and – more importantly – suffers from significant limitations in identifying low emissions in underdeveloped rural areas, and particularly in Africa^34^, which is also – incidentally – a world area highly susceptible to social and armed conflicts.

Accordingly, to address this limitation we create our harmonized nighttime light (NL) indicators using the data generated by Li *et al*.^34^. Briefly, this approach relies on three steps: (i) quantifying monthly VIIRS nighttime light radiance data over the entire continent; (ii) calculating the relationship between VIIRS data and nighttime light emission data from the DMSP/OLS NTL (version 4), which has been included in the PRIO-Grid, exploiting the spatial resolution and radiometric similarities across the two datasets; and (iii) generating consistent nighttime light data by integrating the temporally calibrated DMSP data and DMSP-like data from VIIRS. This results in annual estimates for each 0.08-degree pixel, where the values for each pixel range from 0 (no NL emissions) to 63. We then (iv) aggregate the resulting estimates to the PRIO GRID cell-year level using two approaches to create two distinct indicators.

To create our first NL indicator, (*NL (sum)*), we sum the total levels of nighttime light for each pixel within a given grid cell for each specific year. Considering the number of 0.08-degree pixels within each 0.5-degree grid cell (3600), this results with a wide range of values within the sample (0 ⇔ 220,522). Researchers can divide *NL (sum)* by 3,600 to get a variable measuring average illumination in a given cell-year (with a range of 0 ⇔ 61.26). A major advantage of this approach is that it not only expands the overall temporal coverage from 2014 (used in the PRIO-Grid) to 2018, but also creates a more sensitive nighttime light indicator. The DSMP-like data simulated using VIIRS are better at capturing medium-to-low emissions, making it highly useful for researchers interested in studying the local impacts of development and economic output in Africa, although it is important to stress that researchers should still be careful when applying this indicator to very remote rural areas, considering some uncertainty exists within the lowest luminosity part of the spectrum.

Finally, we create an indicator to approximate annual population densities within each African grid cell. Data for operationalizing this *Population* variable comes from the World Population Counts (WorldPop) dataset, which estimates 0.08 degree (1 km) annual population data between 2000 and 2020 using census data and the method outlined by^35,36^. To create this indicator, we aggregated WorldPop’s mosaiced 1 km data to the cell-year level by summing the total number of people residing in all 0.08 degree pixels recorded within a given grid cell. Again, note that this variable is available solely at the annual cell level due to the temporal availability of WorldPop.

**Fig. 3:**
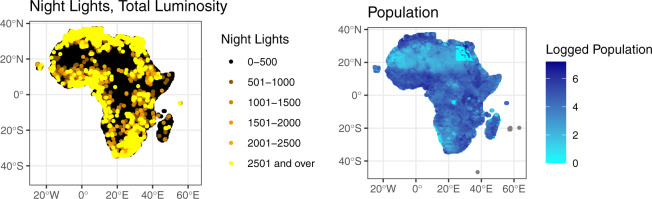
NL (sum) and Population AfroGrid values for June 2011.

For illustration, Fig. 3 plots the values for *NL (sum)* and *Population*, focusing again on June 2011 (due to the annual nature of these indicators, values for June are the same as those for any other month in 2011). In particular, the left plot illustrates the advantages of relying on harmonized NL; this method identifies some level of luminosity over most of the continent, therein improving on DMPS-only series, which tend to underestimate NL emissions in remote rural areas. Moving to the right plot, *Population* levels vary across the continent, with the greatest population concentrations observed in the north, western, and south eastern parts of the continent, and lowest concentrations in deserted areas.

#### Temporal and geographic IDs

As we mentioned in the introduction, to ensure AfroGrid is compatible with other geospatial, event, and country level datasets, we include, temporal, geospatial, and political ID variables to allow researchers to easily incorporate external information. Temporally, we include month and year variables for each grid cell, as well as a combined day-month-year indicator (each day gets a value of 1), to identify the temporal dimension of our panel. Geospatially, we include the longitude and latitude coordinates for each grid cell centroid, as well as each respective grid cell’s ID from the PRIO-GRID dataset. This information allows researchers to integrate any indicator that has geographic coordinates information (e.g., food crops) or integrate such indicators from the PRIO-GRID (e.g., mountains). Politically, we include country ID indicators from the Correlates of War (COW) dataset to allow for the incorporation of relevant country-level features (e.g., democratization, political openness). Using COW IDs is a standard practice in conflict research, but researchers can easily convert these codes to other ID types using R packages such as countrycode. We also include a country name variable. Combined, these different ID indicators allow researchers to incorporate a variety of both time-varying and constant grid- and country-level factors to approximate different confounders and stressors, thereby increasing AfroGrid’s viability for conflict- and non-conflict-related research.

### Measuring social and armed conflict data

To allow researchers to explore the impact of these different issues – and any other indicators of their choice that could be integrated into AfroGrid at the local or country level – on conflict, we operationalize 74 distinct indicators on conflict from four major political violence datasets: (i) the Armed Conflict Location and Event Data (ACLED)^14^ (the ACLED dataset can be accessed at https://acleddata.com/#/dashboard), (ii) the UCDP Geolocated Event Dataset (GED)^15^ (the GED dataset be accessed at https://ucdp.uu.se/downloads/index.html#ged_global), (iii) the Presidential Instability Task Force Worldwide Atrocities Dataset (PITF)^16,37^ (the PITF dataset can be accessed at https://parusanalytics.com/eventdata/data.dir/atrocities.html), and (iv) the Social Conflict in Africa Dataset (SCAD)^17^ (the SCAD dataset can be accessed at https://www.strausscenter.org/ccaps-research-areas/social-conflict/database/). We have received approval from the team behind each dataset to aggregate these data as grid-month indicators. The indicators measure social and armed conflicts along three different dimensions: (i) the perpetrating actor (state forces, rebels, militias, transnational, civilians – including rioters and protesters – and unknown actors); (ii) the type of violence (battles involving armed combatants, fighting between nonstate actors, violence against civilians, riots and protests, etc.), and (iii) the number of fatalities (including lowest, highest, and best estimates), where and when such information was available. Due to space constraints, a discussion of the phenomena and actors measured by each of these variables is provided in the appendix.

Temporally, information is available starting 1989, 1997, and 1995 for the GED, ACLED, and PITF Worldwide Atrocities datasets, respectively, while SCAD data are available over 1990 – 2017 period. Accordingly, AfroGrid includes information going back to January 1989, although we recommend – in order to ensure temporal availability on other variable – to subset relevant years when analyzing relationships between, e.g., NDVI (mean) or NL (sum), and different conflict indicators. Each respective dataset also relies on different thresholds to identify and code a conflict event. For example, the GED records all events that resulted with at least one intentional death. ACLED includes all political violence events, including those that did not involve casualties (ACLED also codes information on other conflict related aspects, such as transfers of territory and strategic development, which we did not integrate into AfroGrid). The PITF only records events that were directed against civilians and caused five or more civilian deaths. SCAD relies on a set of qualitative event definitions.

When aggregating each variable, we included only incidents where information was available at the district or lower (exact coordinates, village) geospatial level and where the exact date, week, or at the very least, month was recorded. This ensures comparability at the grid level as well as a correct temporal aggregation corresponding to AfroGrid’s monthly focus. We also relied on an additional dichotomy when aggregating PITF Worldwide Atrocities data. The PITF Worldwide Atrocities dataset codes two categories of events: (i) incidents, namely distinct atrocity events within a clearly bounded period of time (often at the day level); and (ii) campaigns, a residual category of incidents that last over a longer period, and which usually has lower levels of temporal precision. Accordingly, we aggregate two sets of PITF Worldwide Atrocities indicators into our dataset, one corresponding to incidents, and another corresponding to campaigns.

Additionally, note that SCAD data report information by campaigns, rather than in a temporally disaggregated (e.g., campaign month/week/day) level. We used information on period (month and year) and location to expand this dataset to a campaign-month framework prior to aggregation. To ensure geographic precision, we removed campaigns whose resolution was above the district level; to ensure temporal precision, we removed all campaigns that lasted over one year. This resulted with the removal of 136 campaigns recorded in SCAD, meaning that we were nevertheless able to aggregate more than 86% of the SCAD campaigns into AfroGrid. Note that the focus on the location-month framework when aggregating SCAD into AfroGrid meant that any campaign was recorded only once when aggregated, whether it lasted one day or thirty days. It is important to emphasize, however, that the majority of events recorded in SCAD (73%) lasted only one day, suggesting that any potential aggregation biases are minimal.

Finally, it is important to emphasize that the 74 conflict variables included in AfroGrid are only a subset of all potential political violence indicators that could be created using these four datasets. Moreover, these variables reflect a set of theoretical and empirical choices that – while they were designed to maximize the applicability of these data for climate-conflict analysis as well as broader development-focused research – were subjectively decided upon by the authors. The variables reflect the best data we could obtain at the geographic and temporal resolution levels of interest, which necessitated omitting some events that lacked this information. We made sure to aggregate, whenever possible, data with less precise information, as we did in the case of some SCAD and all PITF campaigns (as opposed to the more specific incidents data). However, we recognize that end users concerned with the potential bias these decisions might prefer a more inclusive approach.

To this end, we included (as mentioned above) the geographic coordinates, PRIO-Grid and country IDs, and month and year indicators for each AfroGrid observation. We also uploaded, as part of our replication data files, all the R scripts used in creating and aggregating each conflict indicator into AfroGrid, to ensure that researchers who seek to create different or more specific (e.g., gender-related violence) conflict indicators can do so easily. This inherent modularity also helps in ensuring relevant new data on conflict and other indicators is constantly integrated into AfroGrid by the users, creating an open/share-source framework that updates over time. In any case, when using AfroGrid’s ‘organic’ conflict indicators, *we ask that you also cite each original dataset* from which the data has been aggregated, to ensure each respective creator team receives its due credit^38–41^.

**Fig. 4:**
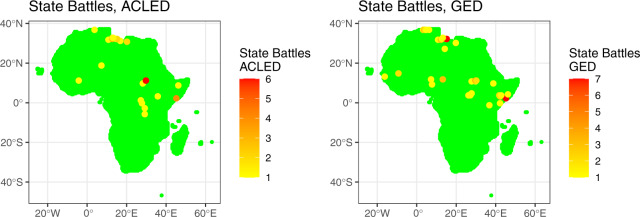
Sample AfroGrid State Based Conflict Indicators (ACLED and GED data) for June 2011.

**Fig. 5:**
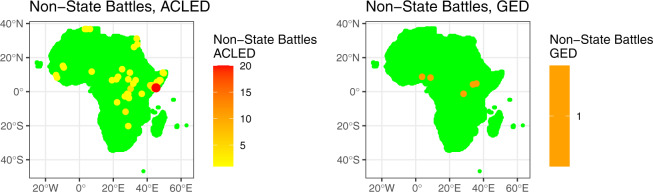
Sample AfroGrid Non-State Based Conflict Indicators (ACLED and GED data) for June 2011.

**Fig. 6:**
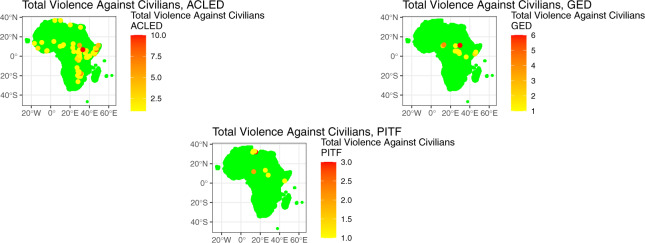
Sample AfroGrid State Based Violence against Civilians Indicators (ACLED, GED, and PITF WAD data) for June 2011.

**Fig. 7:**
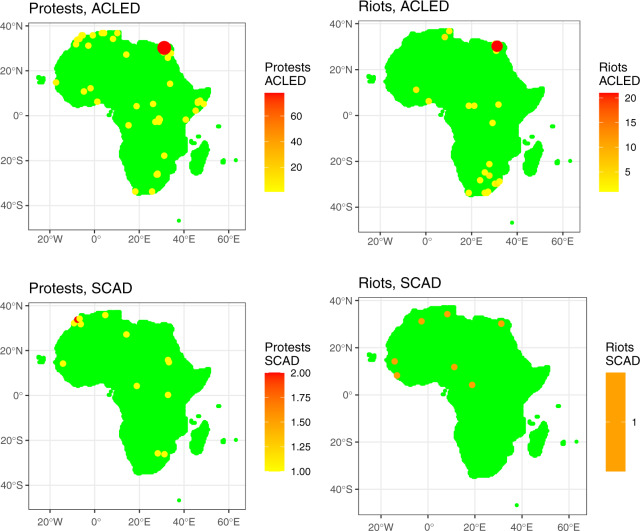
Sample AfroGrid Protests and Riots Indicators (ACLED and SCAD data) for June 2011.

For illustration, Figs. 4–7 plot different collapsed social and armed conflict indicators from all four datasets for the same June 2011 month used in the previous illustrations. Overall, these figures illustrate the advantage of including data on similar phenomena from different analyses within one framework, which can help to ensure that any associated findings are robust. Here, Fig. 4 first plots data from two state-based conflict indicators, *acled_battle_state*_*it*_ and *ged_state*_*it*_. In this case, both datasets seem to identify largely the same subset of relevant state based armed conflict events, although some variability exists. Moving on to Fig. 5, the left plot displays a combined version of several non-state actor violence measures based on ACLED, including attacks by rebels and identity and political militias, while the right plot reports values on fighting involving nonstate actors based on GED data (*ged_nonstate*_*it*_). Here, the divergence in coded events across datasets is more pronounced, and reflects the different substantive definitions (e.g., what actors are included, what casualty threshold is used to code an event) as well as our own choices (e.g., including a variety of nonstate actors when creating the ACLED-based indicator).

Figure 6 then plots violence against civilians from three datasets: ACLED, GED, and PITF Worldwide Atrocities. The plots for ACLED and PITF reflect aggregated values of all violence against civilians indicators by different actors, including the military, rebels, militias, and unknown actors, while the GED plot reflects the dataset’s ‘one-sided violence’ category. Figure 6 shows a noticeable variation in the number and locations of events across the different data, which – again – could be attributed to a variety of reasons (including choice of threshold, qualitative definition of an event, and sources used for measurement).

Finally, Fig. 7 plots peaceful protests and violent riots values from ACLED and SCAD. In both datasets, Cairo is featured prominently, owing to repercussions from the Arab Spring (as the figure represents a snapshot for June 2011). Again, there is some variability across the datasets in terms of the number of events, although this difference could be attributed to the specific approach we took in aggregating SCAD, where campaigns lasting several days were aggregated as a single event, resulting in a potentially more homogenous map. Overall, then, Figs. 4–7 illustrate the importance of comparing any results across different datasets, thereby highlighting the advantage of aggregating conflict indicators from several data projects into AfroGrid.

## Data Records

The aggregated data framework AfroGrid includes all the variables discussed in this note. Variables are measured over the 1989–2020 period, although availability varies for different indicators, but generally information on all variables is available over the 2000–2020 period. The spatial resolution of the data is the 0.5 decimal degree. The temporal unit is the month (excluding nighttime light and population-based variables, which were measured annually and extrapolated to the monthly level). We also included, as a supplementary information file, a list of all these indicators and short summaries of each variable in.pdf format (additionally uploading an.csv version in machine readable form). The uploaded data at the Harvard Dataverse^42^ were compiled in.csv file format. These data can be processed by statistical analysis software such as R and Stata. All scripts and data files can be accessed at: 10.7910/DVN/LDI5TK.

## Technical Validation

As a demonstration of AfroGrid, we analyze different armed and social conflict indicators, using several environmental and developmental determinants, at different levels of temporal aggregation. Here, we first report models where the unit of analysis is the grid cell-month, AfroGrid’s organized panel unit. We then report models where we aggregate all indicators to cell-year level by summing up all conflict indicators to the annual level, and averaging our independent and instrumental variables over the year.

Our conflict dependent variables include all the (aggregated) indicators included in Figs. 4–7, including; from the GED: battles involving state actors, battles involving nonstate actors, and violence against civilians (one-sided violence per GED’s terminology); from ACLED: battles initiated by state actors, and – separately – nonstate actors (aggregating variables related to rebels, identity, and political militias), violence against civilians (aggregating variables for each specific armed actor), protests and riots; from PITF, all atrocities against civilian incidents (aggregating variables for each armed actor); and from SCAD: demonstrations and riots.

For our independent variables, we include *NDVI (mean)* to account for degrees of ‘greenery,’ which can approximate environmental stress; population to account for potential pressures on resources; and *NTL (sum)* to account for local levels of development and state capacity. To account for the time any impacts might take to unfold, we lag our variables by one month (in the cell-month sample) or one year (in the cell-year sample). To account for temporal dependence, we include a one-time-unit lag of each dependent variable, in addition to fixed effects by month and/or year. To account for features that are constant over time, all models include grid cell fixed effects.

Considering the relatively wide spectrum on our conflict variables, we rely on ordinary least squares (OLS) estimators. Additionally, because green coverage might be endogenous to conflict – e.g., because armed troops burn fields and forests – we employ a two-stage least squares (2SLS) estimation strategy. Building on past research (e.g., 4) we use Precipitation (mean) and its quadratic term as instruments to ‘exogenize’ NDVI (mean)’s impact on conflict. Accordingly, each model is identified using the following equations:

1 $${{\bf{y}}}_{{\bf{it}}}={\beta }_{1}\widehat{{{\bf{n}}}_{{\bf{it}}{\boldsymbol{-}}{\bf{1}}}}+{\beta }_{2}{{\rm{In}}{\bf{C}}}_{{\bf{it}}{\boldsymbol{-}}{\bf{1}}}+{\beta }_{3}{{\bf{y}}}_{{\bf{it}}{\boldsymbol{-}}{\bf{1}}}+{\omega }_{{\bf{i}}}+{\tau }_{{\bf{mn}}}+{\tau }_{{\bf{yr}}}+{\in }_{2i}$$

2 $${{\bf{n}}}_{{\bf{it}}{\boldsymbol{-}}{\bf{1}}}={\gamma }_{1}{{\bf{P}}}_{{\bf{i}}{\bf{t}}{\boldsymbol{-}}{\bf{1}}}+{\gamma }_{2}{{\bf{P}}}_{{\bf{it}}{\boldsymbol{-}}{\bf{1}}}^{{\bf{2}}}+{\gamma }_{3}{\rm{In}}\,{{\bf{C}}}_{{\bf{it}}{\boldsymbol{-}}{\bf{1}}}+{\gamma }_{4}{{\bf{y}}}_{{\bf{it}}{\boldsymbol{-}}{\bf{1}}}+{\omega }_{{\bf{i}}}+{\tau }_{{\bf{mn}}}+{\tau }_{{\bf{yr}}}+{\in }_{1i}$$

Here, ***y***_*it*_ is a vector denoting each respective conflict variable, and ***y***_*it-1*_ its lag; ***n***_*it-1*_ is a vector denoting lagged average NDVI values for a given cell-month or cell year; ***p***_*it-1*_ captures lagged precipitation levels in a given cell-month or cell-year; ln(***C***_*it-1*_) are the control variables for population and nighttime light; ***ω***_*i*_ are fixed effects by grid cell; ***τ***_*mn*_ and ***τ***_*yr*_ are fixed effects by month and year, respectively, where the annual level sample models containing only ***τ***_*yr*_; and ε_2i_ and ε_1i_ are the respective error term of each equation.

Note that the models reported in this section represent, for the most part, mostly aggregated versions (in terms of actor and violence type) of the data available in AfroGrid, and that hence the results of these models are not presented as clear conclusions about any specific relationships between environmental stress – or socioeconomic indicators – and armed and social conflict. Nevertheless, Tables 1, 2 illustrate the potential inferential problems that could be caused by relying on the annual instead of monthly level, which is much more inclusive of seasonality.

**Table 1:** 2SLS Regression of Environmental Shocks and Conflict.

	GED						ACLED						PITF	
	Grid month			Grid year			Grid month			Grid year			Grid month	Grid year
	*State (1)*	*Nonstate (2)*	*Violence (3)*	*State (4)*	*Nonstate (5)*	*Violence (6)*	*State (7)*	*Nonstate (8)*	*Violence (9)*	*State (10)*	*Nonstate (11)*	*Violence (12)*	*S. Viol (13)*	*NS. Viol (14)*
$NDVI{\left(\widehat{mean}\right)}_{it-1}$	−0.001 (0.001)	−0.002** (0.001)	−0.002** (0.001)	−0.392 (0.472)	0.592*** (0.118)	0.672** (0.269)	−0.001 (0.002)	−0.001 (0.002)	0.001 (0.002)	−0.528 (0.591)	0.673 (0.504)	3.661*** (0.884)	−0.001 (0.001)	−0.011 (0.129)
*Ln population*_*it-1*_	0.001** (0.0004)	−0.0004 (0.0003)	0.0003 (0.0020)	0.015** (0.004)	−0.003 (0.003)	0.004 (0.004)	−0.001 (0.001)	0.001 (0.001)	0.001 (0.001)	0.0004 (0.006)	0.018* (0.010)	0.013 (0.014)	0.0001 (0.0001)	0.001 (0.001)
*Ln NT (sum)*_*it-1*_	0.00001 (0.00004)	0.00001 (0.00005)	0.00001 (0.00004)	0.001 (0.001)	−0.0003 (0.001)	−0.0002 (0.001)	−0.0002* (0.0001)	0.0002*** (0.0001)	0.0001 (0.0002)	0.002* (0.001)	0.003*** (0.001)	0.0005 (0.002)	−0.00001 (0.00002)	0.0001 (0.0002)
*DV*_*it-1*_	0.466*** (0.107)	0.357*** (0.062)	0.330*** (0.049)	0.419*** (0.021)	0.368*** (0.119)	0.257*** (0.066)	0.623*** (0.121)	0.440*** (0.115)	0494*** (0.062)	0724*** (0.048)	0425*** (0.036)	0549*** (0.060)	0.163*** (0.036)	0.345*** (0.084)
Observations	1,953,816	1,953,816	1,953,816	153,336	153,336	153,336	1,953,816	1,953,816	1,953,816	153,336	153,336	153,336	1,953,816	153,336
R^2^	0.537	0.172	0.186	0.710	0.275	0.339	0.554	0.397	0.429	0.731	0.559	0.596	0.124	0.490
Adjusted R^2^	0.535	0.167	0.181	0.689	0.224	0.291	0.552	0.394	0.426	0.712	0.528	0.567	0.120	0.454

Standard errors clustered on grid cell in parentheses; Ln corresponds to Natural Log; fixed effects by month, year, and grid cell were included in each grid month regression and fixed effects by year and grid cell were included in each grid year regression, although none is reported here.

The instruments used for *NDVI* (*mean*)_*it*−1_ are *Ln precipitation*_*it−1*_ and *Ln precipitation*^2^_*it−1*_. ***p < 0.01, **p < 0.05, *p < 0.1.

**Table 2:** 2SLS Regression of Environmental Shocks and Demonstrations.

	ACLED				SCAD			
	Grid month		Grid year		Grid month		Grid year	
	*Protests (15)*	*Riots (16)*	*Protests (17)*	*Riots (18)*	*Demonstrations (19)*	*Riots (20)*	*Demonstrations (21)*	*Riots (22)*
$NDVI{\left(\widehat{mean}\right)}_{it-1}$	−0.0001 (0.002)	−0.001 (0.002)	−5.374** (2.464)	0.090 (0.850)	−0.001* (0.001)	−0.0001 (0.0004)	−0.360 (0.433)	0.58 (0.176)
*Ln population*_*it-1*_	−0.008*** (0.001)	−0.005*** (0.001)	−0.144*** (0.030)	−0.076*** (0.010)	−0.0004** (0.0001)	−0.0001 (0.0002)	−0.003* (0.002)	0.002 (0.002)
*Ln NT (sum)*_*it-1*_	−0.001*** (0.0002)	−0.001*** (0.0001)	−0.019*** (0.002)	−0.010*** (0.002)	−0.00001 (0.00003)	0.00002 (0.00002)	0.0005 (0.001)	0.0003 (0.0004)
*DV*_*it-1*_	0.506*** (0.056)	0.378*** (0.067)	0.280*** (0.089)	0.327*** (0.069)	0.463*** (0.123)	0.177*** (0.041)	0.420*** (0.042)	0.280** (00.123)
Observations	1,953,816	1,953,816	153,336	153,336	1,831,056	1,831,056	143,106	143,106
R^2^	0.388	0.237	0.393	0.384	0.385	0.093	0.495	0.309
Adjusted R^2^	0.385	0.233	0.350	0.340	0.381	0.088	0.456	0.256

Standard errors clustered on grid cell in parentheses; Ln corresponds to Natural Log; fixed effects by month, year, and grid cell were included in each grid month regression and fixed effects by year and grid cell were included in each grid year regression, although none is reported here.

The instruments used for *NDVI* (*mean*)_*it*−1_ are *Ln precipitation*_*it−1*_ and *Ln precipitation*^2^_*it−1*_. ***p < 0.01, **p < 0.05, *p < 0.1.

For instance, *NDVI (mean)*’s coefficient is negative across all monthly-level GED models, and statistically significant in the latter two models (nonstate actors and violence against civilians). Moving to the annual level models, *NDVI (mean)*’s coefficient is still negative and not statistically significant in the case of state-based conflicts, but it is now positive and statistically significant in the case of battles initiated by nonstate actors and violence against civilians. Moving on to the ACLED columns, similar – although slightly weaker – bias is observed. *NDVI (mean)*’s coefficient in both the monthly and annual ACLED models is negative and statistically insignificant; it is negative in the monthly nonstate actor models but positive (and nearly statistically significant) in the annual model; and it is positive but practically nil in the monthly violence against civilians model, but positive and statistically significant in the annual model. We do not find a similar bias when PITF based indicators are concerned, as the final two columns in Table 1 illustrate.

Moving to Table 2, we find less extensive divergence across ACLED and SCAD based protests and riots indicators, although this could be attributed to the fact that the majority of such events take place in the cities, where greenery levels are generally lower. Nevertheless, it seems the direction, size, and statistical significance of NDVI’s coefficient varies across the levels of temporal aggregation and the sources used to code each dataset.

## Usage Notes

Quantitative research on environmental conflict is gradually recognizing the widespread disconnect between studies across different levels of analysis, time periods, contexts, and actor and violence types. Conflicts, especially those driven related to environmental stress or abundance, are (highly) localized, even though they may have major societal impacts. Recent data work by the teams of ACLED, the GED, PITF Worldwide Atrocities, and SCAD have given us data on a variety of different types of conflict and actors at unprecedented levels of geospatial and temporal resolution, while frameworks such as PRIO-Grid facilitated disaggregating the impact of local determinants at the monthly or annual grid level.

AfroGrid contributed to this ongoing work in several ways. First, by disaggregating local environmental and conflict data to the monthly level at the 0.5-degree resolution, incorporating updated indicators of greenery coverage, temperature, precipitation, and drought, nighttime light, and population data (although in the latter two cases, at the annual level). These indicators are included alongside 74 conflict variables design to facilitate work into linkages on local relationships between the environment, development, and conflict, while also allowing researchers to easily aggregate a variety of indicators at the researcher’s discretion to explore other explanations, as well as how conflict may impact other phenomena. Second, in addition to contributing to quantitative research, AfroGrid also informs the growing qualitative and mixed-method research community, which increasingly complements case studies with quantitative information on climatic factors, nighttime light, and conflict (e.g.^43^,).

These contributions are especially important considering the growing relevance of environmental security research nowadays. Climate-conflict nexus research is regularly published in mainstream journals (e.g^44–46^.), and has been the subject of multiple special issues, appearing in *International Affairs*, *Journal of Peace Resea*rch, and *Politics & Governance* this year (2021) alone. Environmental security issues are now a regular feature of policy debates in the highest levels, including the UN Security Council^47^ and the Pentagon^48^. AfroGrid increases the accessibility of detailed data to investigators of relevant and important questions on these topics, which can inform both research and policymaking.

We would like to end with two notes of caution. First, as mentioned above, in aggregating different armed and social conflict indicators, we made specific decisions about the nature, type, and extrapolation of the data into AfroGrid. As such, we recommend that researchers who deploy AfroGrid will refer to the appendix for a description of these variables and how they were coded, and decide whether these operationalizations make theoretical and empirical sense for their specific analyses. Second, AfroGrid only includes African states and locations. This raises both ethical questions – is it appropriate for researchers primarily located in academic and policy institutions within the Global North to focus so heavily on this world region without appropriate representation of local scholars and policymakers – and empirical questions, especially considering the possibility of the ‘streetlight effect’ and related inferential biases^49^. With respect to the ethical aspect, we are committed to making AfroGrid freely and easily accessible to facilitate the ability of researchers anywhere in the world to access and download the dataset and use it in their own work. With respect to the empirical concern, we suggest researchers should be cognizant of this issue and report any theoretical and empirical findings accordingly. More generally, we hope to extend a similar empirical-aggregational framework to other world regions in the future.

## Supplementary information

Supplemental Information

## Data Availability

Data compilation and aggregation for all indicators was done using R. The complete dataset and R aggregation scripts for the conflict databases are available on the Harvard Dataverse^42^ at: 10.7910/DVN/LDI5TK.
