# Supplementary material for: Introducing AfroGrid, a unified framework for environmental conflict research in Africa
Source: Sci Data. 2022 Mar 29;9:116. doi: 10.1038/s41597-022-01198-5 (PMC8964753; doi:10.1038/s41597-022-01198-5)
Supplement: Supplementary file 1 — Supplemental Information [file 41597_2022_1198_MOESM1_ESM.pdf]

Supplemental Information for:

Introducing AfroGrid, A unified framework for environmental conflict research in Africa

Table of Contents

Conflict indicators 2

GED 2

ACLED 4

PITF 5

SCAD 7

Environmental indicators 8

Socioeconomic indicators 9

ID indicators 9

<u>Conflict indicators</u>

GED

1. *ged_state* – all state-conflict incidents recorded in a given cell month based on information from the GED dataset.
2. *ged_nonstate* – all non-state-conflict incidents recorded in a given cell month based on information from the GED dataset.
3. *ged_viol_tot* – all one-sided violence incidents recorded in a given cell month based on information from the GED dataset.
4. *ged_viol_state* – all one-sided violence incidents perpetrated by a state actor (i.e., an actor recorded as "Government" in the *side_a* column of the GED) recorded in a given cell month based on information from the GED dataset.
5. *ged_viol_nonstate* – all one-sided violence incidents perpetrated by a state actor (i.e., an actor NOT recorded as "Government" in the *side_a* column of the GED) recorded in a given cell month based on information from the GED dataset.
6. *ged_state_fatal_best* – total best fatality estimates for all state conflict incidents recorded in a given cell month based on information from the GED dataset.
7. *ged_state_fatal_high* – total highest fatality estimates for all state conflict incidents recorded in a given cell month based on information from the GED dataset.
8. *ged_state_fatal_low* –total lowest fatality estimates for all state conflict incidents recorded in a given cell month based on information from the GED dataset.
9. *ged_state_fatal_sidea* – total fatality estimates for initiating actor from all state conflict incidents recorded in a given cell month based on information from the GED dataset.
10. *ged_state_fatal_sideb* – total fatality estimates for target actor from all state conflict incidents recorded in a given cell month based on information from the GED dataset.
11. *ged_nonstate_fatal_best* – total best fatality estimates for all non-state conflict incidents recorded in a given cell month based on information from the GED dataset.
12. *ged_nonstate_fatal_high* – total highest fatality estimates for all non-state conflict incidents recorded in a given cell month based on information from the GED dataset.
13. *ged_nonstate_fatal_low* – total lowest fatality estimates for all non-state conflict incidents recorded in a given cell month based on information from the GED dataset.

14. *ged_nonstate_fatal_sidea* – total fatality estimates for initiating actor from all non-state conflict incidents recorded in a given cell month based on information from the GED dataset.
15. *ged_nonstate_fatal_sideb* – total fatality estimates for target actor from all non-state conflict incidents recorded in a given cell month based on information from the GED dataset.
16. *ged_viol_fatal_tot_best* – total best fatality estimates for all one-sided violence incidents recorded in a given cell month based on information from the GED dataset.
17. *ged_viol_fatal_tot_high* – total highest fatality estimates for all one-sided violence incidents recorded in a given cell month based on information from the GED dataset.
18. *ged_viol_fatal_tot_low* – total lowest fatality estimates for all one-sided violence incidents recorded in a given cell month based on information from the GED dataset.
19. *ged_viol_fatal_state_best* – total best fatality estimates for all one-sided violence incidents perpetrated by state actors (as defined in (*4*)) recorded in a given cell month based on information from the GED dataset.
20. *ged_viol_fatal_state_high* – total highest fatality estimates for all one-sided violence incidents perpetrated by state actors (as defined in (*4*)) recorded in a given cell month based on information from the GED dataset.
21. *ged_viol_fatal_state_low* – total lowest fatality estimates for all one-sided violence incidents perpetrated by state actors (as defined in (*4*)) recorded in a given cell month based on information from the GED dataset.
22. *ged_viol_fatal_nonstate_best* – total best fatality estimates for all one-sided violence incidents perpetrated by non-state actors (as defined in (*5*)) recorded in a given cell month based on information from the GED dataset.
23. *ged_viol_fatal_nonstate_high* – total highest fatality estimates for all one-sided violence incidents perpetrated by non-state actors (as defined in (*5*)) recorded in a given cell month based on information from the GED dataset.
24. *ged_viol_fatal_nonstate_low* – total lowest fatality estimates for all one-sided violence incidents perpetrated by non-state actors (as defined in (*5*)) recorded in a given cell month based on information from the GED dataset.

ACLED

25. *acled_battle_state* – all incidents defined as “battles” initiated by state forces recorded in a given cell month based on information from the ACLED database.
26. *acled_battle_rebel* – all incidents defined as “battles” initiated by rebel groups recorded in a given cell month based on information from the ACLED database.
27. *acled_battle_polmil* – all incidents defined as “battles” initiated by political militias recorded in a given cell month based on information from the ACLED database.
28. *acled_battle_idmil* – all incidents defined as “battles” initiated by identity militias recorded in a given cell month based on information from the ACLED database.
29. *acled_remote_state* – all incidents defined as “explosions/remote violence” initiated by state forces recorded in a given cell month based on information from the ACLED database.
30. *acled_remote_rebel* – all incidents defined as “explosions/remote violence” initiated by rebel groups recorded in a given cell month based on information from the ACLED database.
31. *acled_remote_polmil* – all incidents defined as “explosions/remote violence” initiated by political militias recorded in a given cell month based on information from the ACLED database.
32. *acled_remote_idmil* – all incidents defined as “explosions/remote violence” initiated by identity militias recorded in a given cell month based on information from the ACLED database.
33. *acled_viol_state* – all incidents defined as “violence against civilians” initiated by state forces recorded in a given cell month based on information from the ACLED database.
34. *acled_viol_rebel* – all incidents defined as “violence against civilians” initiated by rebel groups recorded in a given cell month based on information from the ACLED database.
35. *acled_viol_polmil* – all incidents defined as “violence against civilians” initiated by political militias recorded in a given cell month based on information from the ACLED database.
36. *acled_viol_idmil* – all incidents defined as “violence against civilians” initiated by identity militias recorded in a given cell month based on information from the ACLED database.

37. *acled_riots* – all incidents defined as "riots" initiated by rioters recorded in a given cell month based on information from the ACLED database.
38. *acled_protests* – all incidents defined as "protests" initiated by protesters recorded in a given cell month based on information from the ACLED database.
39. *acled_fatal_state* – total fatality estimates from all conflict incidents initiated by state forces recorded in a given cell month based on information from the ACLED database.
40. *acled_fatal_rebel* – total fatality estimates from all conflict incidents initiated by rebel groups recorded in a given cell month based on information from the ACLED database.
41. *acled_fatal_polmil* – total fatality estimates from all conflict incidents initiated by political militias recorded in a given cell month based on information from the ACLED database.
42. *acled_fatal_idmil* – total fatality estimates from all conflict incidents initiated by identity militias recorded in a given cell month based on information from the ACLED database.

PITF

43. *pitf_state_inc* – all atrocity incidents perpetrated by state forces recorded in a given cell month based on information from the PITF dataset.[1]
44. *pitf_state_camp* – all new/ongoing atrocity campaigns perpetrated by state forces recorded in a given cell month based on information from the PITF dataset.
45. *pitf_nonstate_sanc_inc* – all state-sanctioned atrocity incidents perpetrated by nonstate actors recorded in a given cell month based on information from the PITF dataset.
46. *pitf_nonstate_sanc_camp* – all state-sanctioned new/ongoing atrocity campaigns perpetrated by nonstate actors recorded in a given cell month based on information from the PITF dataset.
47. *pitf_nonstate_inc* – all unsanctioned atrocity incidents perpetrated by nonstate actors recorded in a given cell month based on information from the PITF dataset.
48. *pitf_nonstate_camp* – all unsanctioned new/ongoing atrocity campaigns perpetrated by nonstate actors recorded in a given cell month based on information from the PITF dataset.

[1] Note that when coding all PITF based indicators, we removed all contested and allegation-based events to ensure we include only substantiated events.

49. *pitf_multiple_inc* – all atrocity incidents perpetrated by multiple state and/or nonstate (pro- and anti-government) actors recorded in a given cell month based on information from the PITF dataset.
50. *pitf_multiple_camp* – all new/ongoing atrocity campaigns perpetrated by multiple state and/or nonstate (pro- and anti-government) actors recorded in a given cell month based on information from the PITF dataset.
51. *pitf_trans_inc* – all atrocity incidents perpetrated by transnational (state and/or nonstate) actors recorded in a given cell month based on information from the PITF database.
52. *pitf_trans_camp* – all new/ongoing atrocity campaigns perpetrated by transnational (state and/or nonstate) actors recorded in a given cell month based on information from the PITF database.
53. *pitf_tot_deaths_inc* – total fatality estimates from all atrocity incidents perpetrated by all actors recorded in a given cell month based on information from the PITF database.
54. *pitf_tot_deaths_camp* – total fatality estimates from all new/ongoing atrocity campaigns perpetrated by all actors recorded in a given cell month based on information from the PITF database.
55. *pitf_state_deaths_inc* – total fatality estimates from all atrocity incidents perpetrated by state forces recorded in a given cell month based on information from the PITF database.
56. *pitf_state_deaths_camp* – total fatality estimates from all from all new/ongoing atrocity campaigns perpetrated by state forces recorded in a given cell month based on information from the PITF database.
57. *pitf_nonstate_sanc_deaths_inc* – total fatality estimates from all state-sanctioned atrocity incidents perpetrated by nonstate actors recorded in a given cell month based on information from the PITF dataset.
58. *pitf_nonstate_sanc_deaths_camp* – total fatality estimates from all state-sanctioned all new/ongoing atrocity campaigns perpetrated by nonstate actors recorded in a given cell month based on information from the PITF dataset.
59. *pitf_nonstate_deaths_inc* – total fatality estimates from all unsanctioned atrocity incidents perpetrated by nonstate actors recorded in a given cell month based on information from the PITF dataset.

60. *pitf_nonstate_deaths_camp* – total fatality estimates from all unsanctioned all new/ongoing atrocity campaigns perpetrated by nonstate actors recorded in a given cell month based on information from the PITF dataset.

61. *pitf_multiple_deaths_inc* – total fatality estimates from all atrocity incidents perpetrated by perpetrated by multiple state and/or nonstate (pro- and anti-government) actors recorded in a given cell month based on information from the PITF dataset.

62. *pitf_multiple_deaths_camp* – total fatality estimates from all new/ongoing atrocity campaigns perpetrated by multiple state and/or nonstate (pro- and anti-government) actors recorded in a given cell month based on information from the PITF dataset.

63. *pitf_trans_deaths_inc* – total fatality estimates from all atrocity incidents perpetrated by perpetrated by transnational (state and/or nonstate) actors recorded in a given cell month based on information from the PITF dataset.

64. *pitf_trans_deaths_camp* – total fatality estimates from all new/ongoing atrocity campaigns perpetrated by perpetrated by transnational (state and/or nonstate) actors recorded in a given cell month based on information from the PITF dataset.

SCAD

65. *scad_org_demo* – all new/ongoing campaigns defined as "organized demonstrations" recorded in a given cell month based on information from the SCAD dataset.

66. *scad_spont_demo* – all new/ongoing campaigns defined as "spontaneous demonstrations" recorded in a given cell month based on information from the SCAD dataset.

67. *scad_viol_riot* – all new/ongoing campaigns defined as "organized violent riot" recorded in a given cell month based on information from the SCAD dataset.

68. *scad_spont_riot* – all new/ongoing campaigns defined as "spontaneous violent riot" recorded in a given cell month based on information from the SCAD dataset.

69. *scad_gen_strike* – all new/ongoing campaigns defined as "general strike" recorded in a given cell month based on information from the SCAD dataset.

70. *scad_lim_strike* – all new/ongoing campaigns defined as "limited strike" recorded in a given cell month based on information from the SCAD dataset.

71. *scad_gov_rep* – all new/ongoing campaigns defined as "pro-government violence (repression)" recorded in a given cell month based on information from the SCAD dataset.
72. *scad_anti_gov* – all new/ongoing campaigns defined as "anti-government violence" recorded in a given cell month based on information from the SCAD dataset.
73. *scad_nsa_viol* – all new/ongoing campaigns defined as "extra-government violence" by non-state groups recorded in a given cell month based on information from the SCAD dataset.
74. *scad_int_gov* – all new/ongoing campaigns defined as "intra-government violence" between two armed factions associated with different elements within the government recorded in a given cell month based on information from the SCAD dataset.

<u>Environmental indicators</u>

75. *NDVI_mean* – the average NDVI value across all 0.08° pixels within a given cell month based on the MODIStsp R package.
76. *NDVI_min* – the lowest NDVI value observed across all 0.08° pixels within a given cell month based on the MODIStsp R package.
77. *NDVI_max* – the highest NDVI value observed across all 0.08° pixels within a given cell month based on the MODIStsp R package.
78. *t.c.avg* – the average temperature across all 0.08° pixels within a given cell month based on the CRU TS monthly high resolution gridded multivariate climate dataset.
79. *t.anom* – the mean deviation (in Z-scored) from the 30-year rolling temperature average for all 0.08° pixels within a given cell month based on the CRU TS monthly high resolution gridded multivariate climate dataset.
80. *p.avg* – the average precipitation across 0.08° pixels within a given cell month based on the CRU TS monthly high resolution gridded multivariate climate dataset.
81. *p.anom anom* – the mean deviation (in Z-scored) from the 30-year rolling precipitation average for all 0.08° pixels within a given cell month based on the CRU TS monthly high resolution gridded multivariate climate dataset.

82. *SPEI – the average SPEI across 0.5°* grid cells, calculated based on temperature and precipitation measures from the CRU TS monthly high resolution gridded multivariate dataset.

Socioeconomic indicators

83. *NL_sum* – total nighttime light emission values (0 ⇔ 63 per pixel) for all 0.08° pixels within a given cell year based on Li et al. (2020).[2]
84. *Population* – the total number of people recorded within all 0.08° pixels in a given cell year based on Li et al. (2020).

ID indicators

85. *month* – the month during which each grid cell was observed.
86. *year* – the year during which each grid cell was observed.
87. *ym* – a year-month-day format, where month corresponds to (86), year corresponds to (87), and the day is the first day of the month (always 1).
88. *latitude* – the latitude of each 0.5-degree cell's centroid.
89. *longitude* – the longitude of each 0.5-degree cell's centroid.
90. *gid – a unique identified for each grid cell assigned in the PRIO-GRID dataset.*
91. *COWCODE* – a three-number designation corresponding to the Correlates of War ID, assigning one cell to one country in any given year (cells were assigned to a country-year based on where the country in which the largest share of their area) using data from the CShapes dataset by Weidmann et al. (2010).[3]
92. *country.name* – the name of the country identified in (92).

[2] X. Li, Y. Zhou, M. Zhao, and X. Zhao, "A harmonized global nighttime light dataset 1992–2018," *Scientific data*, vol. 7, no. 1, pp. 1–9, 2020.
[3] Weidmann, Nils B., Doreen Kuse & Kristian Skrede Gleditsch (2010) The geography of the international system: The CShapes Dataset. International Interactions, 36(1): 86-106.
